# Somatic embryogenesis for mass propagation of elite Spruce families: effect of storage time on somatic embryogenesis initiation

**DOI:** 10.1186/1753-6561-5-S7-P127

**Published:** 2011-09-13

**Authors:** Iftikhar Ahmad, Sofie Johansson, Gisele Andrade, Beata Dedicova, Ulrika Egertsdotter

**Affiliations:** 1 Department of Forest Genetics and Plant Physiology, Swedish University of Agricultural Sciences, Umeå, 90187, Sweden

## Background

Somatic embryogenesis (SE) is the only clonal propagation method that has potential for large scale production of elite conifer plants from the breeding programs. Methods that support bioreactor-based methods for SE propagation are developed [1,2]. Samples of somatic embryos can be stored indefinitely under liquid nitrogen for future plant production. Somatic embryo cultures are also studied as model systems for conifer embryo development to address fundamental research questions, or used as material for genetic transformation to study gene function in conifers.

In addition to utilizing SE for masspropagation of known elite clones previously tested in field tests, the SE technology offers an opportunity to directly capture and increase the value of small samples of elite seeds from the breeding programs. Furthermore, by direct masspropagation of families through SE, the value of the elite seed is increased; however without the cost of clonal testing. This is arguably an alternative approach to the traditional approach of only utilizing clonal field-tested material for SE masspropagation [3].The aim of this project was to investigate the effect from seed storage time on the rate of somatic embryo initiation for the purpose of optimizing the use for SE over time of small valuable seed samples. This was done by isolating ZE from seeds of Norway spruce that had been stored for various times, and were collected from different parts of Sweden.

## Material and methods

### Plant material

Nineteen batches of Norway spruce (*Picea abies*) seeds from commercial seed orchards in southern, middle and northern parts of Sweden were provided by the forest companies supporting the project.

### Initiation of Somatic Embryogenesis

The spruce seeds were sterilized with 95% ethanol followed by 30% (v/v) commercial bleach and Tween 20. The bleach was discarded and the seeds was rinsed three times with sterile distilled water and left to imbibe overnight at room temperature. After imbibition, ZE were dissected from the female gametophyte under a dissecting microscope and cultured on half-strength LP medium supplemented with 10 µM 2, 4-Dichlorophenoxyacetic acid and 4.4 µM Benzyladenine for SE initiation. In total 90 ZE were isolated from each seed batch. The SE initiation was monitored on weekly basis.

### Maturation of Somatic Embryos

One cell line per seed batch was tested for embryo differentiation from pro-embryogenic masses (PEMs) on DKM containing no plant growth regulators (PGRs) and maturation on DKM supplemented with 30 µM Abscisic acid.

## Results

**Figure 1:**
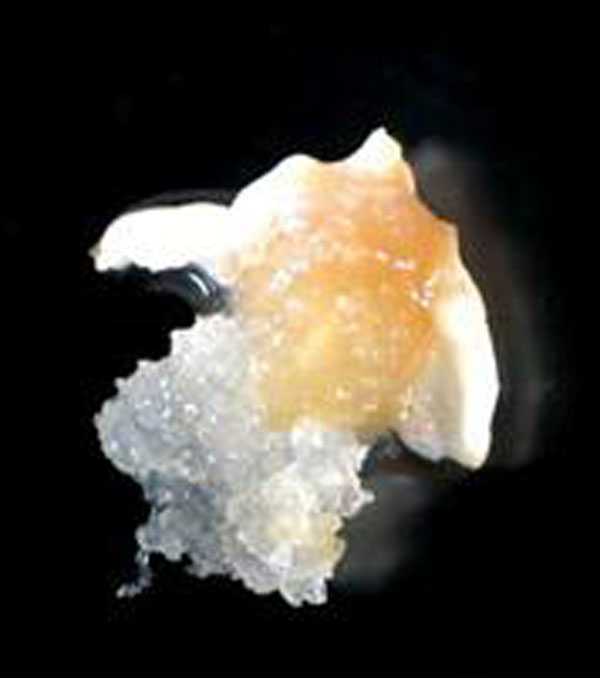
SE initiation from zygotic embryo of Norway spruce. ZE extracted from spruce seeds produced PEMs after 3-4 weeks in culture.

In total we tested 19 seed batches of Norway spruce where 90 ZE were isolated per seed batch and placed on ½ LP medium containing PGRsfor SE initiation. Three weeks after isolation of ZE, callus formation was observed. Embryogenic callus is composed of PEMs that have a white and translucent appearance (Fig. 1) and are mostly produced from the hypocotyl region of the ZE. When whitish callus reached a size of 5x5 mm, it was isolated from primary explants and placed on proliferation medium for continuous growth.

**Figure 2:**
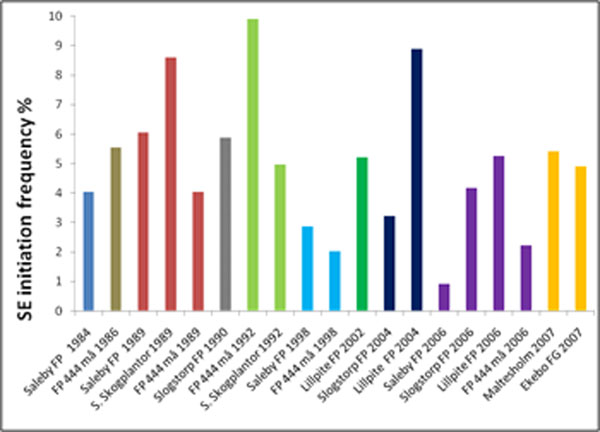
SE initiation rates from seeds from different seed batches and collection years. The SE initiation rate for each seed batch is shown in percentage of seeds tested (90 for each batch) that produced PEMs that could be isolated and cultured. Only one cell line per seed was recorded.

All 19 seed batches showed SE initiation however at different frequencies (Fig. 2). The initiation frequency did not vary notably between the seeds from different parts of Sweden. There was also no difference in initiation frequency related to the time in storage. The seeds tested had been collected between 1984- 2007; the highest initiation frequency was observed in seed batch FP 444 collected in 1992 and the lowest initiation rate was observed in seed batch Saleby collected in 2006.

One of the initiated and established cell lines from each seed batch was subjected to maturation medium to examine whether the cultures of PEMs could produce mature somatic embryos. We observed that 11 out of 19 tested cell lines produced mature somatic embryos (data not shown). Since only one cell line from each seed batch was tested for maturation, we cannot exclude that the remaining 8 seed batches were capable of producing mature somatic embryos. However, similar to the initiation process, the maturation stage did not appear to be related to the storage time and the geographical origin of the seed.

## Conclusion

We have demonstrated that it is possible to propagate small batches of Norway spruce seeds stored for up to 25 years through somatic embryogenesis. All initiated cell lines established cultures of PEMs and most cell lines tested produced mature somatic embryos. Thus we conclude that SE can provide a promising method for amplifying small valuable batches of elite seeds even if the seeds have been stored for up to 25 years.
